# Compression of the Abdominal Aorta

**Published:** 1851-05

**Authors:** Ira E. Oatman

**Affiliations:** Dundee, Ill.


ARTICLE III.

*Compression of the Abdominal Aorta, in a Corpulent Female, with Uterine Hemorrhage, after Delivery.* By IRA E. OATMAN, M. D., of Dundee, Ill.

The practice of compressing the Aorta in uterine hemorrhage I believe, dates no further back, than the time of Baudelocque. It appears strange, that a discovery so simple, and yet so effectual as this, was not made in the days of Harvey. This practice has been adopted and recommended by Chailley, and others. But so far as I have seen, it has only been used in persons of spare, or moderately replete habit. The following case is submitted, to show that it is practicable in corpulent persons, and that it affords means of safety in some cases, in no other way so easily obtained.

On the 19th of November, 1850, I was called early in the morning, to see a lady 26 years of age, in labor with her first child. She was *corpulent*, low in stature, and of the lymphatic, sanguine temperament. The os uteri being little dilated, and the pains inconsiderable, I gave her an anodyne and left her at M. Returning at 4 P. M., I found the pains had been suspended for an hour, but they had returned slightly, and were more frequent. They continued to increase in strength slowly and tardily, the interval being about eight minutes. At 10 ½ P. M., she was delivered of a small male child. The uterus contracted slightly and the placenta came away in ten or fifteen minutes, with an unusual quantity of blood. The uterus did not remain contracted, but became so flaccid, that it could not be felt through the walls of the abdomen.

I depressed the head and shoulders of the patient and gave her ergot and plumb. acet., in doses as large as the stomach would bear. I continued the efforts through the parietes of the abdomen, to excite the uterus to contraction, which only occurred slightly, and in about eight minutes, as before delivery. I applied pounded ice over the abdomen.

The os uteri was still dilated, flaccid and occluded with coagula. The pulse was indistinct in the radial artery, the countenance was pale, anxious and ghastly, and the lips retracted. She had intense thirst and indiscribable debility, with occasional syncope. Fearing that she would die before I could remove the coagula, and use the hand for an internal stimulant to the womb; or use cold astringent injections, I directed my efforts to the abdominal aorta as a dernier resort, notwithstanding the excessive fatness of the patient.

After considerable ineffectual effort, I found by making fearfully hard pressure, wiih the ends of the fingers, about two inches above the umbilicus, that the pulsations of the aorta could be controlled, this being the only eligible point for the operation. This gave instantaneous relief to all the urgent and fatal symptoms. I continued this pressure during three hours and ten minutes. The womb continued flaccid. Being compelled to change the hands often, sufficient blood would pass down to prevent the open mouths of the uterine vessels from being occluded, with dense coagula. Hence, I thought it unsafe to discontinue the pressure sooner. Although the uterus was little contracted at the end of this time, the hemorrhage did not return. During this period the most perfect quiet was observed, the ice continued, opii, plumb. acet and ergot was given occasionally ; and she was often allowed rich chicken tea, which she took with great avidity.

The pulse slowly and gradually returned. By observing quiet and using nutricious drinks, with a little wine, she had no other untoward symptoms. After a few days I gave her small doses of sul. quinia, and ferri ferro-cyan., and acid nitric. She gradually and permanently recovered her usual health. The child did well.

I have had one case since, in which it was necessary to resort to compression of the aorta, in menorrhagia, after delivery. This also was with a first child, but the lady being of a spare habit, it required but little skill to practice it successfully.

Dundee, March 23, 1851.

